# Regression Model Fitting With Quadratic Term Leads to Different Conclusion in Economic Analysis of Washington State Smoking Ban

**Published:** 2010-12-15

**Authors:** Marshal Ma, Scott McClintock

**Affiliations:** Pennsylvania Department of Health, Harrisburg, Pennsylvania; West Chester University, West Chester, Pennsylvania

## To the Editor:

Authors of a recent article studied the economic effect of a 2006 smoking ban on bars and taverns in Washington State (1). Their findings of higher-than-expected taxable sales in bars and taverns could have a broad influence on future policy decisions in other states that still do not have these laws.

We found some issues with the authors' methods. The authors used taxable retail sales (TRS) data from 2002 through 2007 to fit the following regression model:

ln(TRS_bar)_*i*_ = b_0_ + b_1_SFL_*i*_ + b_2_Q2_*i*_ + b_3_Q3_*i*_ + b_4_Q4_*i*_ + b_5_t_*i*_ + b_6_SFL_*i*_ t_*i*_+ b_7_UNEMP_*i*_ + b_8_lnPOP_*i*_ + b_9_lnINC_*i*_ + e_*i*_

Based on the raw TRS data provided to us by the authors (Table 1) the taxable retail sales from 2002 to 2005 or 2007 do not seem to follow a linear trend over time. Instead, the overall trend from 2002 to 2005 or 2007 seems to be parabolic. Thus, the quadratic model is a better fit for the data than the linear model (Figure).

**Figure 1:**
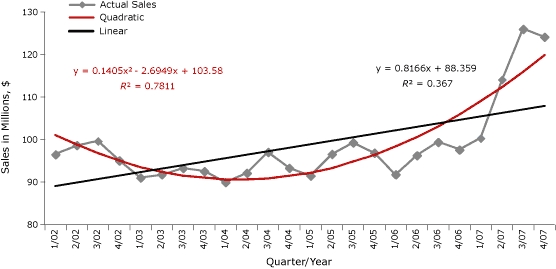
Comparison of regression fit to taxable retail sales in bars and taverns in Washington State after the implementation of a smoke-free law, from the first quarter of 2002 (1/02) through the fourth quarter of 2007 (4/07). Values are adjusted for inflation to the Consumer Price Index (www.bls.gov/cpi/). The figure displays 2 regression equations for the quadratic fitting (y = 0.1405x^2^ − 2.4969x + 103.58, R^2^ = 0.7811) and the linear fitting (y = 0.8166x + 88.359, R^2^ = 0.367) .

**Table:** 

**Quarter/Year**	**Sales in Millions, $**	**Linear**	**Quadratic**
1/02	96.5	89.2	101.0
2/02	98.7	90.0	98.8
3/02	99.7	90.8	96.7
4/02	95.1	91.6	95.0
1/03	91.1	92.4	93.6
2/03	91.9	93.3	92.5
3/03	93.3	94.1	91.6
4/03	92.6	94.9	91.0
1/04	90.0	95.7	90.7
2/04	92.2	96.5	90.7
3/04	97.1	97.3	90.9
4/04	93.4	98.2	91.5
1/05	91.5	99.0	92.3
2/05	96.6	99.8	93.4
3/05	99.3	100.6	94.8
4/05	96.9	101.4	96.4
1/06	91.8	102.2	98.4
2/06	96.3	103.1	100.6
3/06	99.4	103.9	103.1
4/06	97.6	104.7	105.9
1/07	100.3	105.5	108.9
2/07	114.1	106.3	112.3
3/07	126.0	107.1	115.9
4/07	124.2	108.0	119.8

We fit the TRS data provided by the authors to a regression model with a quadratic term as well as a model without a quadratic term. Although the variables unemployment, population, and income were unavailable, we used all other variables in our model. The model with the quadratic term provided a better fit (*R* ^2^ = 0.95) than the model without the quadratic term (*R* ^2^ = 0.89). Also, with the quadratic time term in the model, the time by smoking ban interaction is no longer significant, which suggests that the smoking ban did not affect the taxable sales revenue over the time. This result contradicts the authors' conclusion that the smoking ban had an effect on taxable retail sales. Our model, without the quadratic term, predicts a $98.5 million increase during the 2 years after implementation of the smoke-free law. This is close to the $105 million predicted by the authors, and the difference is presumably accounted for by the absence of unemployment, population, and income variables in our model. However, if we include the quadratic term our model shows a $42.3 million decrease in taxable retail sales during the 2 years after implementation of the smoke-free law (Table 2). Inclusion of a quadratic term leads to considerably different conclusions.

## Figures and Tables

**Table 1:** Quarterly Taxable Retail Sales in Bars and Taverns,^a^ Washington State, 2002-2007

**Quarter/Year**	Sales in Millions, $
1/02	96.5
2/02	98.7
3/02	99.7
4/02	95.1
1/03	91.1
2/03	91.9
3/03	93.3
4/03	92.6
1/04	90.0
2/04	92.2
3/04	97.1
4/04	93.4
1/05	91.5
2/05	96.6
3/05	99.3
4/05	96.9
1/06	91.8
2/06	96.3
3/06	99.4
4/06	97.6
1/07	100.3
2/07	114.1
3/07	126.0
4/07	124.2

a Adjusted to the Consumer Price Index (www.bls.gov/cpi/).

**Table 2:** Difference in Projected Sales,^a^ Using a Model With and Without Quadratic Term, in Bars and Taverns, Washington State, 2006-2007

**Quarter/Year**	Difference Without Quadratic Term	Difference With Quadratic Term
1/06	4.9	10.9
2/06	-0.1	9.3
3/06	-5.0	7.7
4/06	-9.9	6.1
1/07	-14.8	4.5
2/07	-19.7	2.9
3/07	-24.6	1.3
4/07	-29.5	-0.3
**Total**	-98.5	42.3

a Values represent the difference between projected quarterly taxable retail sales with smoking ban and without smoking ban, in millions of dollars, adjusted to the Consumer Price Index (www.bls.gov/cpi/).
